# Current Treatment and Immunomodulation Strategies in Acute Myocarditis

**DOI:** 10.1097/FJC.0000000000001542

**Published:** 2024-05-02

**Authors:** Emma Ferone, Amitai Segev, Erika Tempo, Piero Gentile, Ahmed Elsanhoury, Chiara Baggio, Jessica Artico, Prashan Bhatti, Paul Scott, Emanuele Bobbio, Marco Merlo, Pietro Ameri, Gianfranco Sinagra, Carsten Tschöpe, Daniel Bromage, Antonio Cannata

**Affiliations:** * School of Cardiovascular Medicine and Sciences, King's College London, London, United Kingdom;; † Cardiovascular Division, Chaim Sheba Medical Center, Tel Hashomer, Ramat-Gan, Israel;; ‡ The Sackler Faculty of Medicine, Tel-Aviv University, Tel Aviv, Israel;; § Department of Internal Medicine, University of Genova, Genova, Italy;; ¶ Ospedale Niguarda Ca'granda, Milan, Italy;; ‖ Berlin Institute of Health (BIH) Center for Regenerative Therapies (BCRT), Berlin, Germany;; ** Department of Cardiology, Angiology, and Intensive Medicine (CVK), German Heart Center at Charite (DHZC), Berlin, Germany;; †† German Centre for Cardiovascular Research (DZHK), Partner Site Berlin, Berlin, Germany;; ‡‡ CardioThoracoVascular Department, Azienda Sanitaria Universitaria Giuliano-Isontina, Trieste, Italy;; §§ Department of Cardiology, Hammersmith Hospital, Imperial College Healthcare NHS Trust, London, United Kingdom;; ¶¶ Institute of Cardiovascular Science, University College London, London, United Kingdom;; ‖‖ King's College Hospital NHS Foundation Trust, London, United Kingdom;; *** Department of Cardiology, Sahlgrenska University Hospital, Gothenburg, Sweden;; ††† Department of Molecular and Clinical Medicine, Institute of Medicine at Sahlgrenska Academy, University of Gothenburg, Gothenburg, Sweden;; ‡‡‡ Cardiovascular Disease Unit, Cardiac, Thoracic and Vascular Department, IRCCS Ospedale Policlinico San Martino, Genova, Italy; and; §§§ Institute of Heart Diseases, Wroclaw Medical University, Wroclaw, Poland.

**Keywords:** myocarditis, steroids, immunosuppression, treatment, inflammation, inflammatory disease

## Abstract

Myocarditis is an inflammatory disease of the myocardium characterized by a great heterogeneity of presentation and evolution. Treatment of myocarditis is often supportive, and the evidence for immunosuppression is scarce and debated. Conventional treatment is based on clinical presentation, ranging from conservative to advanced mechanical assist devices. In this setting, immunosuppression and immunomodulation therapies are mostly reserved for patients presenting with major clinical syndromes. In this review, we will summarize the current evidence and strategies for conventional and immunosuppressive treatments for patients presenting with acute myocarditis.

## INTRODUCTION

### Definitions

**FIGURE 1.**
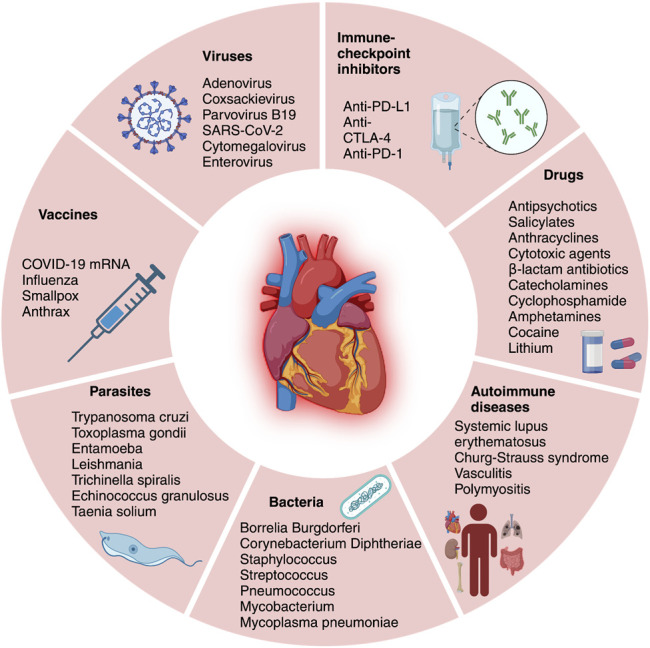
Different forms and etiology of myocarditis.

Myocarditis is a disease caused by inflammation of the myocardium and cardiomyocyte necrosis. The etiology includes viral, bacterial, and parasites infections^1^ or activation of the immune system by autoimmune diseases, drugs, or vaccines.^1–3^ In the absence of a universally accepted definition of myocarditis, several different terms and definitions have been described^2,4^ (Fig. 1). Contemporary definitions include the World Health Organization definition, where myocarditis is defined as an inflammatory condition requiring histological, immunological, and immunohistochemical evidence of inflammation associated with nonischemic cardiomyocyte damage.^5,6^ However, other definitions specific to the etiology, infiltrate, and clinical presentation have also been proposed.^2^

### Epidemiology

The exact incidence of myocarditis is difficult to estimate because of varying definitions of myocarditis. The most recent Global Disease Study (GBD 2019) estimated an annual incidence of 16 cases per 100,000 worldwide.^7^ Myocarditis was defined as an acute inflammatory condition of the heart, and cases were identified from health records containing a diagnosis of myocarditis.^7,8^ Because the accuracy of electronic health care records to detect myocarditis is low and the definition is broad,^9^ it is likely that myocarditis is both under-reported and over-reported in different regions and depending on the criteria used.

There is, however, consensus that myocarditis has a higher incidence in male patients, which is as high as 19 cases per 100,000 men per year compared with 13 cases per 100,000 women per year, usually occurring in the third and fourth decade of life,^7,10^ with the highest risk being between 20 and 40 years.^1,8^ Treatments such as immune checkpoint inhibitors (ICI) and autoimmune diseases are also associated with an increased risk of developing myocarditis.^2^

### Initial Presentation

Clinical presentation is heterogeneous. It includes chest pain, dyspnea, fatigue, palpitations, arrhythmias, and syncope.^2,10,11^ Patients may also report prodromal symptoms suggestive of preceding viral infection, although their absence does not exclude myocarditis.^2,11^ Life-threatening arrhythmias or left ventricular (LV) systolic dysfunction,^2^ cardiogenic shock,^1,2,11^ and sudden cardiac death may also be the initial presentation.^11^ In a recent retrospective observational study in the United Kingdom, patients presenting with chest pain had better prognosis compared with patients presenting with dyspnea or arrhythmias.^10^

## DIAGNOSIS

There are several challenges to the diagnosis of myocarditis. Endomyocardial biopsy (EMB) remains the gold standard for diagnosis.^11^ The Dallas criteria are used to confirm myocarditis using EMB histopathology results.^6^ Advantages of EMB include the ability to classify myocarditis based on the infiltrating cell type (lymphocytic, eosinophilic, giant cell myocarditis [GCM], granulomatous).^2^ However, EMB is limited by the invasive nature of the test, which can cause complications.^2,12^ Furthermore, patchy myocardial involvement reduces its sensitivity. Repeated EMB may be indicated particularly if clinical suspicion for GCM is high,^13^ in which case immunosuppressive therapy is recommended.^14^ Further analysis of EMB specimens using viral polymerase chain reaction genome analysis, which may reveal the presence of infection, may indicate it is safe to initiate targeted therapies such as immunosuppression or antiviral therapy.^11^ Active viral replication should be ascertained, where possible, before commencing immunosuppressive treatment. It should include viruses associated with myocarditis such as enteroviruses, parvovirus B19, HHV-6, human cytomegalovirus, hepatitis C virus, adenovirus, and Epstein–Barr virus.^15^ To date, there are no randomized control trials available investigating the benefits and safety of immunosuppression in cases with an active cardiotropic viral infection.^16^ Therefore, the use of immunosuppression is this setting is still debated and based on preclinical and observational evidence, showing that immunosuppression in myocarditis with enterovirus genome presence may be associated with worse outcomes.^17^ However, the role of persistent viral genome is debatable.^18^ In high-risk cases, such as fulminant myocarditis or GCM, immunosuppressive treatment may be administered while awaiting viral genome analysis results^19^ because the benefits may outweigh the risks. Specific antiviral therapies, such as interferon-β, may also be considered for adenovirus, enteroviruses, or in cases of HHV-6.^15^

Cardiac magnetic resonance imaging is increasingly available as an adjunct to EMB. It allows tissue characterization and scar quantification. It has greatest utility when performed between 2 and 3 weeks from symptom onset.^2^ It should be carefully evaluated when performed within than 4 days from symptom onset because late gadolinium enhancement may appear later after presentation in some forms of myocarditis.^20^ The diagnosis of myocarditis based on cardiac magnetic resonance imaging uses the Lake Louise Criteria, which require evidence of both edema on T2-weighted image or mapping findings as well as myocardial injury seen on late gadolinium enhancement, T1 mapping, and extracellular volume.^21^

Echocardiography findings suggestive of myocarditis may include increased wall thickness and mild segmental hypokinesia.^2^ The presence pericardial effusion further supports the diagnosis of myocarditis.^11^ In addition, LV systolic function is a strong predictor of outcome^2,22,23^ and may be useful to guide patient management, although the use of ejection fraction to guide therapy has not been confirmed in randomized studies.

Biomarkers include myocardial necrosis biomarkers such as high-sensitivity troponin and creatinine kinase-MB and nonspecific inflammatory biomarkers such as C-reactive protein and erythrocyte sedimentation rates.^2^ Raised biomarkers are nonspecific, but they support the clinical suspicion and may be elevated in at least 60% of patients.^10,22,24–26^ Abnormal myocardial necrosis biomarkers, such as troponin I or T, are helpful for a diagnosis of acute myocarditis (AM) because if within the normal range, the diagnosis of an acute myocardial injury is unlikely.^26^ Abnormal white blood counts may indicate the etiology of the myocarditis, such as eosinophilia in eosinophilic myocarditis (EM).^2^ Virology swabs^2,11^ and serum autoantibodies may also be useful in identifying etiology.^11^

Several diagnostic criteria have been proposed for diagnosis of clinically suspected myocarditis.^11^ These include diagnostic and clinical presentation criteria. Clinical suspicion of myocarditis should prompt further investigations. While these criteria are useful for highlighting cases which may benefit from more invasive investigations, they have not yet been validated. The lack of universal diagnostic criteria for AM remains a challenge. This heterogeneity of clinical *Consensi* makes accurate comparisons between studies challenging.^9^

## INITIAL TREATMENT

**FIGURE 2.**
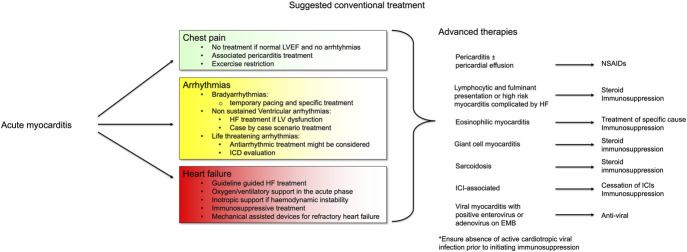
Etiology-guided treatment of myocarditis.

**FIGURE 3.**
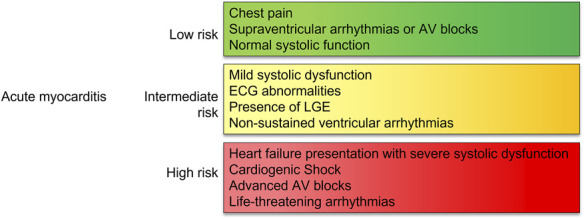
Risk stratification in myocarditis.

Supportive management is the mainstay of therapy for patients presenting with AM.^11^ Further management of myocarditis may include immunosuppression with steroids.^11^ Empirical immunosuppression has, so far, failed to demonstrate a strong clinical benefit^27^ and is only recommended in patients with GCM or after exclusion of active viral replication at EMB in the most severe patients.^1,2,11,14,28^ Risk stratification in AM is important (Figs. 2, 3). Indeed, studies investigating the role of immunosuppression in myocarditis were generally small and conducted in selected high-risk populations. Other anti-inflammatory therapies that have been investigated for use in myocarditis include nonsteroidal anti-inflammatory drugs (NSAIDs), colchicine, monoclonal antibodies, and other immunosuppressants such as azathioprine (AZA) and methotrexate (MTX). This review aims to summarize available treatments for myocarditis (Fig. 2), discuss challenges with the existing evidence base, and review emerging novel therapies.

## CONVENTIONAL TREATMENTS

The management of myocarditis includes nonspecific measures to treat the *sequelae* of heart disease, including heart failure (HF) therapy and treatment of arrhythmias according to current guidelines, as well as the use of etiology-specific therapy when indicated.

Asymptomatic or low-risk patients may require admission for monitoring or management of symptoms.^11^ Hemodynamically stable patients with LV systolic dysfunction should be managed according to general HF guidelines.^1,11^ Medical treatment relies on early initiation of guideline-directed therapy including angiotensin-converting enzyme inhibitors, angiotensin receptor blockers or angiotensin receptor–neprilysin inhibitors, beta-blockers, mineralocorticoid receptor antagonists, and sodium–glucose cotransporter 2 inhibitors and appropriate use of diuretics. Although clinical evidence is currently limited for AM, the use of these drugs is recommended based on expert consensus and animal studies suggesting potential benefits.^1,11,29–33^ In patients with myocarditis and normal LV systolic function, the initiation of medical therapy is not recommended.

Management of hemodynamically unstable patients should be conducted in an intensive care unit with respiratory and mechanical cardiopulmonary support facilities, and referral to a specialized tertiary care center should be considered. More severe presentations of myocarditis, such as cardiogenic shock or hemodynamically unstable LV dysfunction, require intensive care unit admission and vasopressor, inotropic^1^ or mechanical circulatory support.^11^ Ventricular assist devices and extracorporeal membrane oxygenation as bridge to recovery or transplantation^2,11^ may also be useful in patients who present with severe ventricular dysfunction refractory to medical therapy.^25,34,35^ These devices can provide hemodynamic stabilization while minimizing the risk of inotrope-induced arrhythmias and allowing time for the heart to recover.^28,36,37^ After 2–3 weeks without successful weaning from mechanical circulatory support, consideration should be given to the possibility of long-term LV assist device or heart transplantation.^2^

Management of conduction disturbances, such as atrioventricular block and ventricular arrhythmias, is usually supportive because these arrhythmias tend to resolve after the acute phase. However, the use of pacemakers or antiarrhythmic drugs may be required in some cases. Myocarditis secondary to Lyme disease, ICI, or sarcoidosis can present with advanced conduction abnormalities in patients with normal or near-normal LV function.^38–40^ Treatment of arrhythmias and conduction disturbances in patients with myocarditis has no specific recommendations, and the management of these conditions in the postacute phase should follow existing guidelines on arrhythmia and device implantation.

Implantable cardioverter-defibrillator implantation is typically not recommended during the acute phase of myocarditis because the risk of arrhythmia may subside within the following 3–6 months. During this time, a wearable cardioverter-defibrillator may serve as an alternative, although there is limited evidence supporting this strategy.^41^ However, in a subset of patients with a high arrhythmic risk, implantable cardioverter-defibrillator implantation may be fast-tracked to reduce the arrhythmic risk.^42^

Physical activity should be restricted during the acute phase of myocarditis until the disease has completely resolved. This recommendation is based on evidence of increased viral replication in the heart with exercise compared with controls in murine models of coxsackievirus infection^43^ but is not supported by robust evidence in humans. The optimal duration of exercise restriction is uncertain, but avoiding moderate-intensity to high-intensity exercise between 3 and 6 months from the index event is usually suggested.^44^ Before clearance, patients may be evaluated with a symptom-limited exercise test, Holter monitor, and echocardiogram.^45,46^

## NSAIDs

Clinical data on the effect of NSAIDs, such as aspirin, ibuprofen, and indomethacin, in myocarditis are limited and controversial.^1,11,47^ In animal models, the use of NSAIDs in AM may cause additional myocardial damage^48–51^; however, data are lacking in humans. However, in the context of myopericarditis or perimyocarditis, where the pericardial involvement is more pronounced,^52–54^ the use of NSAIDs is safer, particularly in those patients with a preserved left ventricular ejection fraction (LVEF).^54^ Nowadays NSAIDs are mainly used in low-risk myocarditis, presenting with pericardial chest pain, florid inflammation, and without LV dysfunction. In these cases, the treatment regimen can be similar to that used in acute pericarditis.^53^ Conversely, in high-risk myocarditis complicated by HF, the use of NSAIDs may be harmful.^55^ Therefore, an immunosuppressive strategy, using high doses of corticosteroids or an association of corticosteroids with other immunosuppressive agents such as AZA, mycophenolate mofetil, or cyclosporine, may be preferred.^11,47^

## COLCHICINE

The anti-inflammatory agent, colchicine, traditionally used to treat acute gouty arthritis,^56^ has recently been shown to improve cardiac function in different inflammatory cardiac disorders,^57–59^ including pericarditis with pericardial effusion.^60–62^ EMB samples of patients with myocarditis showed an amplified expression of NLRP3 inflammasome and related cytokines including interleukin (IL)-1β and IL-18, reflecting greater myocardial injury.^63–65^ These processes are targeted by colchicine, through its reduction of superoxide production and inhibition of inflammasomes and IL-1β production.^63^ The principal mechanism of colchicine is stalling microtubule polymerization, which disrupts the cytoskeleton and cell division. This significantly alters neutrophil functions including chemotaxis, adhesion, and mobilization. In addition, colchicine has antifibrotic and endothelial-protective features.^63^ Although limited evidence is available, colchicine may be useful in patients with myocarditis by targeting the underlying inflammatory processes.^62^

Colchicine also improves cardiac function and reduces the inflammasome 3 activity in cytomegalovirus B3 (CVB3)–induced myocarditis mice model.^66^ Interestingly, this mechanism decreases cardiac and splenic NLRP3 inflammasome activity, without exacerbation of CVB3 load.^66^ A proof-of-concept placebo-controlled, randomized multicenter study investigating the efficacy of colchicine in inflammatory cardiomyopathy is underway (EU Trial Number: 2023-503350-12-00). Nevertheless, further large-scale clinical trials are necessary to prove the efficacy of colchicine for myocarditis treatment.

## IMMUNOSUPPRESSION WITH STEROIDS

The role of corticosteroids in AM is controversial. Only 1 randomized clinical trial has assessed the efficacy of immunosuppression for AM.^67^ In this trial, Mason et al studied the effect of prednisone with either cyclosporine or AZA in 111 patients with a histopathological diagnosis of myocarditis and a LV ejection fraction less than 45%. The immunosuppressive protocol did not significantly improve mortality compared with conventional HF therapy. However, the treatment was administered between 2 weeks and 2 years after the clinical presentation, resulting in the potential inclusion of cases of long-standing chronic nonischaemic cardiomyopathy secondary to myocarditis. Furthermore, a genetic background, which is present in up to 30% of patients with AM, may have influenced the results^68^ because immunosuppressive treatment may have blunted effects in established dilated cardiomyopathy.^6^ Another similar trial, the TIMIC trial, investigated the effect of immunosuppression in chronic inflammatory cardiomyopathy.^69^ Unlike Mason et al, the TIMIC trial obtained results in favor of immunosuppression. This may be attributable to the exclusion of a viral infection on EMB or the targeting of a different stage of the immune response.^69^ As cases associated with viral infections were not excluded by Mason et al,^67^ beneficial effects of immunosuppression may have been attenuated by the hazardous effects of immunosuppression during a cardiotropic viral infection,^69^ although the effects of immunosuppression on viral myocarditis remains unknown.

While immunosuppression is strongly recommended in specific noninfectious myocarditis settings, such as GCM, EM, cardiac sarcoidosis (CS), and immune checkpoint inhibitor-associated myocarditis, the role of steroids is debated in the other scenarios.^70^ The most recent expert consensus document on management of AM and chronic inflammatory cardiomyopathy^2^ suggests consideration of empirical intravenous (IV) corticosteroids in cases of fulminant myocarditis or complicated AM, tailoring the therapy according to EMB results. However, considerable divergence remains between official recommendations and clinical practice, including the possibility of starting immunosuppressive therapy empirically, without knowing viral polymerase chain reaction results on EMB.^19^ This is supported by the finding that viruses, particularly PVB-19 and HHV-6, may be found in a large proportion of patients who do not have myocarditis.^16,65,71^ Ongoing clinical trials are assessing the role of high-dosage methylprednisolone in the context of AM complicated by HF or fulminant presentation (MYTHS, NCT05150704)^72^ and potentially may expand the indication for steroids in patients with AM.

## NONCONVENTIONAL IMMUNOSUPPRESSANTS

Several nonconventional forms of immunosuppression have been proposed or are under investigation to determine their potential benefits in the context of AM. These include recombinant cytokine receptor antagonists such as anakinra^73–75^ or lymphocyte components involved in the inflammatory process in myocarditis. Noncorticosteroid immunosuppressants including AZA,^69,76,77^ MTX,^78^ and cyclosporine^67^ have also been proposed for use in the management of myocarditis.

### Anakinra and Other Monoclonal Antibodies

The key involvement of IL-1 in acute inflammation and the observation of elevated levels of this cytokine in AM support the use of IL-1–targeting therapies for this condition.^79^ Case reports and case series have suggested that anakinra, a recombinant human IL-1 receptor antagonist, may promote LV function recovery in both adults and children with lymphocytic AM.^73,74^ More robust evidence on treatment of AM with anakinra comes from the Anakinra versus Placebo for the Treatment of Acute Myocarditis (ARAMIS) trial, which is now completed and has compared anakinra in addition to standard therapy versus standard therapy alone in admitted patients with AM (NCT03018834).^75^ Results from the ARAMIS trial showed that Anakinra is safe for use but does not reduce the complications of myocarditis in a relatively low risk population.^80^ Further studies are required to elucidate the role of Anakinra in myocarditis.

In specific subsets of AM, other monoclonal antibodies have been used, often alongside corticosteroids or as a second-line therapy after corticosteroid failure.^81^ Alemtuzumab, which targets CD52 on B and T cells and is indicated for multiple sclerosis, has shown potential efficacy in ICI-related AM.^82^ Muronomab has successfully been used in ICI-related myocarditis and in GCM.^83^ It targets CD3, a glycoprotein found on T cells, and is already approved in organ transplant recipients. Abatacept, a CTLA-4 antagonist, also holds promise in ICI-related myocarditis,^84^ while the administration of anti–IL-6 monoclonal antibodies, ie, tocilizumab and sarilumab, has yielded conflicting results.^85,86^

Anti–IL-5 monoclonal antibodies may have a role in the management of EM. Mepolizumab reduces the occurrence of flares in hypereosinophilic syndrome (HES) and was approved by the Food and Drug Administration for the treatment of eosinophilic granulomatosis with polyangiitis (EGPA) in 2017, particularly in asthmatic patients.^87,88^ In a phase 2 trial involving symptomatic patients with FIP1L1-negative and PDGRFA-negative hypereosinophilia, benralizumab was associated with lower absolute eosinophil counts and long-term sustained response as compared with placebo.^89,90^

Infliximab is a chimeric IgG1 monoclonal antibody that binds tumor necrosis factor alpha with high affinity, neutralizing its proinflammatory actions. Based on expert consensus, antitumor necrosis factor alpha agents are currently recommended as a third-line therapy for severe refractory sarcoidosis and relapsing/remitting EGPA.^91,92^

Although the role of CD20-expressing B lymphocytes in cardiac impairment in rheumatologic diseases is debated, rituximab, a chimeric monoclonal anti-CD20 antibody, has shown effectiveness in systemic lupus erythematous-related myocarditis and in recurrent idiopathic GCM after heart transplantation.^93–95^

### Noncorticosteroid Immunosuppressants and Immunotherapies

AZA and MTX inhibit purine and pyrimidine synthesis, thereby halting the proliferation of inflammatory cells. Cyclosporine is a calcineurin inhibitor that blocks the synthesis of interleukins, including IL-2, which is essential for self-activation and differentiation of T lymphocytes. Mycophenolate mofetil depletes guanosine nucleotides preferentially in T and B lymphocytes, hindering their expansion.

All these immunosuppressants have been considered for treatment of myocarditis. However, their administration in AM lacks solid evidence from clinical trials and remains controversial, particularly when viral genome is detected in the myocardium because immunosuppression might favor viral spreading and, therefore, direct cardiomyocyte damage.^11,64^ On the other hand, the presence of a virus may be the trigger of a primarily immune-mediated AM, without causing significant myocardial injury per se.^16^ Despite these concerns, immunosuppressant may be warranted in cases of fulminant AM such as GCM, as discussed above, where clinical deterioration can be dramatically rapid and prompt treatment is necessary. In this context, T-cell–targeting agents are typically combined with high-dose corticosteroids.^35,96^

In chronic myocarditis, especially associated with immune-mediated systemic diseases, immunosuppressants are commonly used as maintenance therapy and as corticosteroid sparing agents. A notable example is the utilization of MTX and AZA in CS.^97^ The effectiveness of this approach is currently being investigated in the CS Randomized Trial (CHASM-CS-RCT) (NCT03593759).^78^

Three randomized controlled trials have been conducted with immunosuppressants in myocarditis, of which 1 enrolled patient with AM did not find differences in LVEF and survival between conventional therapy and conventional therapy plus AZA or cyclosporine.^67^ The other 2 recruited subjects with chronic inflammatory cardiomyopathy led to discordant results (Table 1).^76,98^ The Study to Evaluate the Efficacy of Immunosuppression in Myocarditis or Inflammatory Cardiomyopathy (IMPROVE-MC) is further testing AZA together with corticosteroid in biopsy-proven virus-negative AM or inflammatory cardiomyopathy (NCT04654988).^77^

**TABLE 1.** Summary of Epidemiology of Myocarditis Etiologies

Etiology	Incidence	Associated Conditions	Inclusion Criteria/Cohort	References
Lymphocytic myocarditis	95.5% of EMB confirmed myocarditis cases		Patients undertaking an EMB between 1983 and 2010 for clinically suspected myocarditis or nonischaemic cardiomyopathy Left, right, or biventricular EMB-proven myocarditis	McNamara et al^99^
Giant cell myocarditis	0.007%^100^	Hashimoto thyroiditis, Crohn disease^2,12^ ulcerative colitis, polymyositis, vasculitis, systemic lupus erythematosus, Sjogren syndrome^101^	Autopsy records between 1958 and 1977	Schultheiss et al^100^
Eosinophilic myocarditis	3.6%^99^	Hypersensitivity, eosinophilic granulomatosis with polyangiitis, and parasitic infections^2^ Peripheral eosinophilia^101^ 60% of patients with hypereosinophilic syndrome^102^	Patients undertaking an EMB between 1983 and 2010 for clinically suspected myocarditis or nonischaemic cardiomyopathy Left, right, or biventricular EMB-proven myocarditis	McNamara et al^99^
ICI-myocarditis	1.14%	Cancer treatment with immune checkpoint inhibitors	Patients receiving ICI treatment between November 2013 and July 2017	Tschöpe et al^103^

Cyclophosphamide pulses have been used alongside corticosteroids for EM associated with EGPA.^92^ Nevertheless, there are no data to support the notion that the addition of cyclophosphamide to corticosteroids improves outcomes in patients with AM.

Finally, high-dose intravenous immunoglobulins (IVIG) have been proposed for treatment of AM by virtue of their immunomodulatory and anti-inflammatory activities. Two clinical trials have assessed IVIG in AM/inflammatory cardiomyopathy but have been inconclusive^99,104^ (Table 2). McNamara et al^99^ did not require evidence of inflammation as part of the inclusion criteria; therefore, inclusion of cases with a noninflammatory cause of dilated cardiomyopathy may have resulted in equivocal findings. Differences in IVIG treatment regimens may also account for inconclusive results. McNamara et al report that nearly half of the IVIG group received the total dose over 4 days rather than 2 days as used in the remaining patients in the IVIG group and by Kishimoto et al.^99,104^ Therefore, further work is required to identify an IVIG drug regimen for optimal immunosuppression.

**TABLE 2.** Clinical Trials With Immunosuppressants in Myocarditis/Inflammatory Cardiomyopathy

References	No. of patients	Treatments	Key Inclusion Criteria	Duration	Outcome
Mason et al^61^	111	Conventional therapy + cyclosporine or AZA versus conventional therapy	Histopathological diagnosis of myocarditis and LVEF <45%	28 wk	No significant difference in LVEF change and survival between the 2 groups
Frustaci et al^74^	85	AZA + prednisone versus placebo	CHF of unknown cause with LVEF <40% and biopsy-proven myocardial inflammation	6 mo of treatment, 2 yr of follow-up	Increase of LVEF in the treatment group (vs. decrease in the placebo group)
Wojnicz et al^72^	84	Standard therapy (diuretic, ACEi, beta-blocker) + prednisone and AZA versus placebo	CHF of unknown cause with LVEF <40% and biopsy-proven myocardial inflammation	3 mo of treatment, 2 yr of follow-up	No significant difference in the primary composite end point (death, heart transplantation, and hospital readmission) between the groups Significant increase of LVEF in the treatment group after 3 mo, persisting at 2 yr of follow-up
Kishimoto et al^95^	41	15 pts treated with high-dose IVIG	LVEF ≤40% and no more than 6 mo of cardiac symptoms at the time of randomization	2 mo	Significant difference in survival between patients receiving versus not receiving IVIG
McNamara et al^96^	62	Conventional therapy + IVIG versus conventional therapy	Recent-onset (<6 mo) dilated cardiomyopathy with LVEF <40% with or without inflammation on biopsy	2 yr	No significant difference in LVEF change between the 2 groups

ACEi, angiotensin-converting enzyme inhibitor; CHF, chronic heart failure.

## ANTIVIRAL THERAPY

In the rare cases of viral positivity on EMB specimens, the usefulness of antiviral therapy is still not well established and there are insufficient data to support it.^1^ Only little available evidence has shown beneficial effects of interferon treatment on viral clearance and New York Heart Association functional class for chronic inflammatory cardiomyopathy related to enterovirus and adenovirus.^100,101^ The use of antiherpetic drugs has been proposed in patients with myocarditis and HHV-6, Epstein–Barr virus, or cytomegalovirus infection, although their efficacy is still unproven.^11,102,103^ Telbivudine and IVIG may have a potential therapeutic role, but more studies are needed to confirm their efficacy.^103^

Finally, no evidence is currently available regarding the combination of antiviral and immunosuppressive therapy in virus-positive inflammatory cardiomyopathy. Hence, in these rare clinical contexts, it is reasonable to involve an infectious disease specialist before deciding on specific antiviral treatment.

## SPECIFIC ETIOLOGY

### Giant Cells Myocarditis

GCM is a rare, yet often fatal form of myocarditis if not treated adequately.^14^ Myocardial damage mediated by CD4^+^ T lymphocytes and infiltration of giant cells, eosinophils, and macrophages results in LV dysfunction and ventricular arrhythmias.^2,14,105^ Prompt initiation of immunosuppression remains key to the management of GCM, with the consideration of transplantation in select cases.^14^ Treatment with conventional immunosuppression (such as AZA, prednisone, and cyclosporine) with addition or substitution of mycophenolate mofetil, muromonab, and MTX has been shown to provide transplant-free clinical remission in up to 65% of patients with GCM.^13^ The addition of mycophenolate treatment to immunosuppressive therapy has also been shown to aid recovery of GCM.^13,96,106^ In fulminant cases, case reports suggest antithymocyte globulin or muromonab combined with high-dose IV methylprednisolone and cyclosporine, a calcineurin inhibitor, may be beneficial.^2,107^

### Eosinophilic Myocarditis

EM is rare but life-threatening. The reported in-hospital mortality rate is approximately 17%.^108^ EM is often associated with Churg–Strauss syndrome, HES, or EGPA, and treatment may vary according to the underlying cause and associated conditions.^2^ In cases of hypersensitivity reactions, prompt identification and cessation of the causative drug cessation of the causative drug is recommended.^2^ Corticosteroids have been shown to reduce the in-hospital mortality of EM not associated with hypereosinophilic syndromes; however, evidence is limited to a meta-analysis of observational data.^108^ Overall, case reports show high-dose corticosteroids were used to successfully treat all forms of EM.^109–115^ In cases associated with EGPA, cyclophosphamide, AZA, rituximab, and mepolizumab were also used.^113–115^ A case of EM with Churg–Strauss syndrome was treated with combined high-dose corticosteroids and cyclophosphamide, whereas in EM associated with HES, only prednisone or methylprednisolone were used.^109–112^ Owing to the uncommon nature of the condition, there are no trials for the management of EM; therefore, evidence is limited to case reports, meta-analyses, and retrospective case studies.^2^

### Cardiac Sarcoidosis

Sarcoidosis is an inflammatory disease of unknown etiology with multisystem involvement. Symptoms of cardiac involvement of sarcoidosis have been reported in 3%–43% of patients with systemic disease.^116^ CS is characterized by the formation of non-necrotizing granulomas within myocardial tissue, as well as chronic fibrosis and inflammation.^2,116^ AM due to sarcoidosis is typically treated with corticosteroids.^2,116^ However, specific regimens and strategies for steroids are largely empirical given the lack of randomized clinical trials in this setting. Initial treatment with prednisone is recommended; however, the dose of corticosteroid immunosuppression should be adjusted according to the severity of symptoms. Life-threatening presentations such as cardiogenic shock, malignant arrhythmias, and widespread inflammation on imaging may warrant a stronger corticosteroid therapy or the addition of a second immunosuppressant.^116^ Although 2 small studies have suggested combined therapy to be more favorable, robust evidence is lacking.^117,118^ MTX is considered as a second-line therapy for patients with sarcoidosis and myocarditis or as an alternative for patients who cannot tolerate them.^2^ Targeting the pathological release of IL-1, using the IL-1 antagonist Anakinra, is another approach being explored to treat CS in a novel pilot study.^119^ Anakinra is expected to dampen the inflammatory response and safely reduce systemic inflammation.^120^ AZA, leflunomide, cyclophosphamide, and biologics such as infliximab and rituximab have been previously used for CS management.^116,121^ Response to therapy should be monitored and includes, but is not limited to, 12-lead electrocardiogram, cardiac biomarkers, and functional imaging.^116^

### ICI-Myocarditis

ICI have revolutionized the landscape of cancer therapy, but their use may be complicated by immune-related adverse events. Among these, myocarditis is the most severe complication. The clinical suspicion often arises after clinical symptoms onset and an increase in cardiac biomarkers or electrocardiographic manifestations.^122^ Current guidelines on the management of ICI-associated cardiovascular toxicity recommend rapid initiation of high-dose oral or IV corticosteroids and withdrawal of ICI as first-line therapy for symptomatic patients.^123^ High-dose methylprednisolone, alemtuzumab (anti-CD52 antibody), antithymocyte globulin (anti-CD3 antibody), and abatacept (a CTLA-4 agonist) have been proposed as second-line therapy for patients without an immediate response to initial treatment.^2,123^ Despite treatment, mortality of ICI myocarditis remains high.^2,123^

## COVID-19 AND VACCINE-ASSOCIATED MYOCARDITIS

### COVID-19–Associated Myocarditis

During the period March 2020 to January 2021, great attention has been paid to SARS-CoV-2 infection–associated myocarditis. In some reports from electronic health care records, COVID-associated myocarditis accounted for up to 41% of cases, with an incidence of all-cause myocarditis increased to 150 cases per 100,000 people during this period.^124^ However, these data should be interpreted with caution as a direct pathogenic link has not been demonstrated yet. Management of COVID-19–associated myocarditis follows the same recommendations as those for viral myocarditis, which may therefore include steroids.^125^

### COVID-19 Vaccine–Associated Myocarditis

The incidence of COVID-19 vaccine–associated myocarditis was reported to peak at 105.9 cases per million in male patients between 15 and 17 years.^126^ Symptomatic management and conventional HF treatment (if required) remain central to the management of vaccine-related myocarditis.^126^ However, in the absence of rigorous studies to recommend the use of steroids, dexamethasone has been proposed as potential therapy because of its previous use in myocarditis.^127^

## FUTURE PERSPECTIVES

**FIGURE 4.**
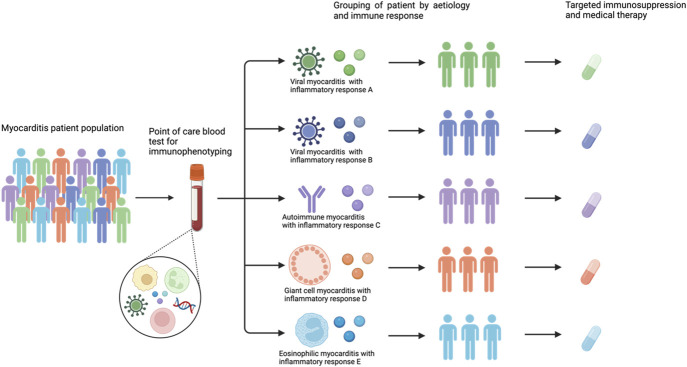
Future of personalized medicine in myocarditis.

The evidence base for the treatment of myocarditis remains controversial, and there is no consensus on optimal treatment strategies for these patients (Table 3). This is likely to be due, in part, to heterogeneous pathophysiology and might justify why “one-size-fits-all” immunosuppression has had limited success to date. For example, viral myocarditis, which is associated with PAMPs and the activation of innate immunity,^4^ may respond differently to autoimmune myocarditis, which is believed to be characterized by T-lymphocyte–mediated adaptive immune responses.^4,11,128^ While studies are ongoing in specific etiologies, this is often unknown, especially at the time of initial clinical presentation or where EMB is not readily available or represents an unacceptable risk to the patient. Therefore, future efforts should be directed at understanding immune phenotypes and relevant, point-of-care biomarkers to guide targeted anti-inflammatory and immunosuppressive therapies. This is possible with contemporary, multiomics approaches. For example, RNA-seq and pathway enrichment analysis identify upregulated pathways in GCM.^129^ These included genes associated with neutrophil degranulation, cytokine signaling, and phagocytosis. Using such approaches, including at single cell resolution,^130^ is likely to identify novel therapeutic targets and allow tailoring of existing medical therapy to a patient's particular immune environment (Fig. 4).

**TABLE 3.** Commonly Reported Immunosuppression Regimens According to Etiology of Myocarditis

Etiology	Mostly Suggested Treatment	Other Considerations	Further Treatments	Evidence
Viral (inflammatory cardiomyopathy associated with enterovirus and adenovirus)	Interferon-β-1b 4 or 8 million IUs every other day for 24 wk (up titration from 2 million IUs every other day during week 1)		—	Randomized clinical trial^116^
Lymphocytic acute (presenting with acute HF/fulminant manifestation)	Initial IV pulses of methylprednisolone (500–1000 mg for 3 d) and maintenance at 1 mg/kg could be considered on individual bases			
Giants cell^2,70^	Methylprednisolone 1 g daily for 3 d Taper at 1 mg/kg/d to 5–10 mg/d at 6–8 wk End therapy after 1 year or continue 5 mg/daily indefinitely and cyclosporine BID (target trough levels, 150–250 ng/mL) and azathioprine 1.5–2 mg/kg/d for 1 yr In refractory/severe cases: Antithymocyte globulin 100 mg IV daily for 3 d or alemtuzumab 30 mg IV for 1 d or 15 mg IV for 2 d	—	Tacrolimus (target trough levels, 5–15 ng/mL [10–15 ng/mL in first 6 month, 5–10 ng/mL thereafter]) Mycophenolate mofetil 1.5 g BID for 1 yr Rituximab 375 mg/m^2^ once a week for 4 wk, continue once every 4 mo	Expert consensus statement^70^
Eosinophilic (hypersensitivity reaction, eosinophilic granulomatosis with polyangiitis, and myeloproliferative variant of HES)	Methylprednisolone IV 1 g/d for 3 d Then continue with prednisone orally 1 mg/kg/d Prednisone 40 mg orally, taper over 28 d	Withdrawal of suspected drug in case of hypersensitivity reaction	Cyclophosphamide IV 600 mg/m^2^ on day 1, 15, and 30 or Rituximab 375 mg/m^2^ weekly × 4 (number of cycles ranging 1–4) or anti-IL-5 agents: mepolizumab, 100–300 mg SC/4 wk, or benralizumab, 30 mg SC/4–8 wk or Albendazole, 600–800 mg/d, for 2–8 wk or Imatinib, 100–400 mg/d, for 4–28 d (up to normalization of eosinophilic count)	Case reports^71^
ICI-associated^84,128^	Methylprednisolone IV 500–1000 mg/d for 3 d initiated within 24 h of symptom onset or Prednisone 1–2 mg/kg/d oral or IV	Withdrawal of immune checkpoint inhibitor therapy	Abatacept IV, 10–25 mg/kg, on days 0, 5, and 12 or ATG IV, 1 mg/kg, usually single dose or Alemtuzumab IV, 30 mg, single dose or Ruxolitinib, 10–15 mg, BID, for 2–4 wk	
Cardiac sarcoid^77^	Prednisone 0.5 mg/kg/d. Titrate prednisone down every 4 wk, reducing dose by 5–10 mg until a maintenance dose of 5–10 mg Methylprednisolone IV 500–1000 mg/d for 2–3 d		Methotrexate 10–25 mg/wk or azathioprine 1–2 mg/kg/d Infliximab IV 5 mg/kg at week 0, 2, 4 and then every 8 wk for 1 year or until inflammation resolves	Case reports and expert consensus

ATG, anti-thymocyte globulin; BID, 2 times a day; JACC, Journal of American College of Cardiology; QDS, 4 times a day; SC, subcutaneous; TDS, 3 times a day.

## CONCLUSIONS

Myocarditis is a heterogenous condition. Treatments of myocarditis are often supportive, and evidence in the field is scarce. Immunosuppression has been debated for decades in this setting, with conflicting results. Nonsteroidal anti-inflammatories, antiviral agents, steroids, and nonsteroidal immunosuppressants have, however, important roles in specific forms of myocarditis. Overall, the evidence base for treatment remains mixed and requires a collective focus on deep immunophenotyping of patients with myocarditis. In the era of personalized medicine, a targeted approach through novel multiomics techniques may help to identify novel targets and upregulated inflammatory pathways to facilitate targeted treatment of patients with myocarditis.
